# Design and Implementation of Respiration Rate Measurement System Using an Information Filter on an Embedded Device

**DOI:** 10.3390/s18124208

**Published:** 2018-11-30

**Authors:** Radius Bhayu Prasetiyo, Kyu-Sang Choi, Gi-Hun Yang

**Affiliations:** 1 University of Science and Technology, Daejeon 34113, Korea; jq_rbp@yahoo.com; 2 Robotics Group, Korea Institute of Industrial Technology, Gyeonggi-do, Ansan-si 15588, Korea; 3 Manufacturing System Group, Korea Institute of Industrial Technology, Chungcheongnam-do, Cheonan-si 31056, Korea; kschoi@kitech.re.kr

**Keywords:** Photoplethysmography (PPG), respiration rate, information filter

## Abstract

In this work, an algorithm was developed to measure respiration rate for an embedded device that can be used by a field robot for relief operations. With this algorithm, the rate measurement was calculated based on direct influences of respiratory-induced intensity variation (RIIV) on blood flow in cardiovascular pathways. For this, a photoplethysmogram (PPG) sensor was used to determine changes in heartbeat frequencies. The PPG sensor readings were filtered using an Information Filter and a fast Fourier transform (FFT) to determine the state of RIIV. With a relatively light initialization, the information filter can estimate unknown variables based on a series of measurements containing noise and other inaccuracies. Therefore, this filter is suitable for application in an embedded device. For faster calculation time in the implementation, the FFT analysis was calculated only for a major peak in frequency domain. Test and measurement of respiration rate was conducted based on the device algorithm and spirometer. Heartbeat measurements were also evaluated by comparing the heartbeat data of the PPG sensor and pulse-oximeter. Based on the test, the implemented algorithm can measure the respiration rate with approximately 80% accuracy compared with the spirometer.

## 1. Introduction

In recent years, rapid technological advances have led to significant developments in the field of robotics, especially for medical purposes [1]. From the microscopic scale to a large robotic surgery system, these developments have brought significant progress in treatment and health-care system [2,3,4,5]. Robots are also becoming more portable and compact as they are needed for assessing first aid in dangerous environments that cannot be accessed by personal medical help [6,7,8,9,10,11,12,13]. Furthermore, robots need to be able to locate and preliminarily determine the physical state of an injured person so that a correct course of treatment can be quickly administered by the medical help. Therefore, a medical sensory system that can determine the physical state must be implemented in a robotic system, especially one that is related to vital health conditions, such as heartbeat and respiration rate.

The respiration rate is a vital biosignal state that determines the physical state of the pulmonary system, which directly influences blood flow in cardiovascular pathways. Due to this, the measurement of blood flow in cardiovascular pathways can reflect a heartbeat condition as inhale and exhale activities can directly increase or decrease blood flow in those pathways [14,15,16,17]. Therefore, by measuring the changes in blood flow or heartbeat frequencies in the cardiovascular stream, the respiration rate can also be determined. The parameter of respiratory-induced intensity variation (RIIV) can also be analyzed based on these changes [15,16,17,18,19,20,21,22]. One technique for detecting these flow changes and has been widely used to measure blood oxygen saturation is photoplethysmography (PPG).

PPG is a method that requires an optical measurement to detect blood volume changes in the body caused by the cardiovascular system [14,15,16,17,18,19,20,21,22]. The PPG measurement can be achieved by employing a light source and a photodetector. Red or near-IR light-emitting diode (LED) with a wavelength of more than 600 nm is commonly used as the light source for the PPG sensor. This LED works as a transmitter that illuminates the skin tissue and is captured by the photodetector. The variations of light intensity in the skin detected by the photodetector can be associated with changes in blood volume in the captured area. Therefore, using this noninvasive method, the PPG sensor can be used to measure the blood flow rate caused by the cardiovascular system that reflects the heartbeat condition.

Some PPG-based methods have been studied for estimating respiration rate and applied to a smartphone-camera-acquired pulse photoplethysmographic (SCPPG) signal [23]. Derivation of the respiration rate signal in [23], was conducted by observing variations of PPG data signal that was acquired from a smartphone’s camera, i.e., SCPPG signal. Similar with [14,15,17,22], the SCPPG signal was then filtered using a passband filter. Non-pulse-to-pulse methods based on amplitude and frequency modulation sequences, were also tested to derive the respiration signals from the filtered data. Furthermore, combination of the PPG-based (pulse-to-pulse) and non-pulse-to-pulse methods were also studied to extract respiration rate information from the SCPPG signal. The study in [23] suggests that derivation of normal ranges of respiration rates can be obtained from SCPPG signal with reasonable accuracies.

This development in the PPG signal processing have broadened application of the PPG sensor in healthcare monitoring system. Furthermore, with the sensor’s portability and relatively small in size, the sensor can also be used and implemented into a small embedded device, such as a wearable health device. This wearable device is commonly equipped with multiple sensor modules to monitor physical condition of its user, such as body temperature, heartbeat, respiration rate, and motion. The device also needs a special patch or strap to be placed on the user body or skin. As an example, the wearable health device developed by Byteflies, Sensor Dot, can measure multiple vital signals, e.g., photoplethysmogram (PPG), electrocardiogram (ECG), respiration, motion, electrodermal activity (EDA), and electromyogram (EMG) [24]. The device utilizes multiple sensor signals with a multimodal signal analysis so that it can provide a whole new level information for medical assessment. These powerful features are very useful for general health-care monitoring system, especially that for complex analysis of physical state determination.

In this work, an algorithm for measuring the respiration rate was developed for an embedded device. Unlike previous study [23], an information filter is implemented to the embedded device for filtering data from a PPG sensor. The device is attached to a field robot for relief, search and rescue operations. Additionally, to be able to provide first aid in a critical situation, the robot carries other medical instruments, such as syringe and oxygen delivery system. Furthermore, the robot must be able to locate the victims and preliminarily determine their physiological states, such as their heartbeat, respiration rate, and body temperature. For that, the robot will deploy the embedded device from a safe compartment after removing obstructions in measurement area. Therefore, the device must be small, lightweight, low-powered, and can be easily deployed with minimum measurement requirements and procedures. Then to minimize redundant data and/or computation process while keeping simplicity in design, the embedded device only utilized one PPG sensor module for heartbeat and respiration rate measurement. Likewise, the algorithm should not be too complicated and can be run in such limited resources with reasonable reliability. Here, the algorithm was implemented to an embedded device with dimension of about 50 × 40 × 30 mm and a weight of about 40 grams. Additionally, the embedded device can measure heartbeat and respiration rate with a relatively simple procedure, i.e., by slightly pressing it to skin or human body.

## 2. Respiration Rate Measurement Algorithm

The algorithm for respiration rate measurement was derived from raw data readings (i.e., the heartbeat signal) of the PPG sensor. This data was then converted to a digital number (discretization) by an analog-to-digital converter (ADC) system in the embedded device. Then, a PPG signal was derived based on the output value of this ADC system. The signal was then filtered using information filter to remove noises in the PPG signal during the readings and discretization process.

The information filter or inverse covariance filter, is a well-known filter that has same function and procedures as the Kalman filter [25]. With a relatively light initialization, the information filter can estimate unknown variables based on a series of measurements containing noise and other inaccuracies. Therefore, this filter can produce optimal parameter estimation of the state and observation model from measurements with Gaussian noise, such as in object tracking systems [25,26,27].

Same as Kalman filter, the information filter procedure consists of initialization, propagation, and update [25]. This procedure was applied to two components of the information filter, i.e., inverse of mean (${\hat{y}}_{k|k}$) and inverse of covariance ($P_{k|k}^{-1}$). Since its components are in an inverted state, propagation in information filter is more complex than that in Kalman filter, but the update is simpler. Additionally, similar with Kalman filter, the information filter propagates and updates the two components when measurement inputs arrive.

Here, the information filter takes the PPG signal as a measurement input and track its value $a_{k}$ and rate of change $b_{k}$ from time k−1 to time k ($T_{k|k-1}$), as shown in Equation (1). Then, variable F can be described as a motion function of the PPG signal changes during that time, as shown in Equation (2):

(1) $$\left[\frac{a_{k}}{b_{k}}\right]=F\left[\frac{a_{k-1}}{b_{k-1}}\right]$$

where:

(2) $$F=\left[\begin{array}{cc} 1 & T_{k|k-1}\\ 0 & 1 \end{array}\right]$$

In the initialization step, the information filter sets value of ${\hat{y}}_{0|0}$ and $P_{0|0}^{-1}$ to zero. With this, information filter will accept all of the measurement inputs that arrive later, at time k = 1. Then, the filter will use all of that inputs to propagate and update its components. Thus, compared with Kalman filter that needs covariance in a big number during initialization, the information filter actually has relatively light setup.

(3) $${\hat{y}}_{k|k-1}={\left(I+AQ\right)}^{-1}F^{-T}{\hat{y}}_{k-1|k-1}$$

(4) $$P_{k|k-1}^{-1}={\left(I+AQ\right)}^{-1}A$$

where:

(5) $$I=I_{2}$$

(6) $$A=F^{-T}P_{k-1|k-1}^{-1}F^{-1}$$

(7) $$Q=qdef\left[\begin{array}{cc} \frac{T_{k|k-1}^{4}}{3} & \frac{T_{k|k-1}^{3}}{2}\\ \frac{T_{k|k-1}^{3}}{2} & T_{k|k-1}^{2} \end{array}\right]$$

(8) qdef=0.375

Equations (3) and (4) describe propagation of the information filter. In here, I is an identity matrix 2 × 2 and Q is calculated based on Equation (7) with qdef equals to 0.375. In Equations (3) and (4), matrix Q is used to introduce uncertainties of sampling time fluctuation and discretization error to the motion function in Equation (2). In Equation (7), variable $T_{k|k-1}$ corresponds to sampling time fluctuation and qdef is related to discretization error. In here, variable qdef is derived based on differences of PPG signal data based on ADC reading and smoothed line approximation in every sampling time equal to 1. Then based on average of the differences, optimum value of qdef can be estimated, i.e., equals to 0.375. Variable ${\hat{y}}_{k|k-1}$ is an inverse of mean that is propagated from time k−1 to time k. The ${\hat{y}}_{k|k-1}$ is directly proportional with inverse of transpose of variable F, i.e., $F^{-T}$. Likewise, $P_{k-1|k-1}^{-1}$ is the propagation result of the inverse covariance.

After the propagation and when the PPG sensor captures a heartbeat signal at a given time k, the information filter updates its components by using Equations (9) and (10). In this update, the values of matrices H and R are configured to reduce noise and avoid non-linearity from the PPG signal, as shown in Equations (11) and (12):

(9) $${\hat{y}}_{k|k}={\hat{y}}_{k|k-1}+H^{T}R^{-1}z_{k}$$

(10) $$P_{k|k}^{-1}=P_{k|k-1}^{-1}+H^{T}R^{-1}H$$

where:

(11) $$H=\left[\begin{array}{cc} 1 & 0\\ 0 & 0 \end{array}\right]$$

(12) $$R=\left[\begin{array}{cc} 50 & 50\\ 50 & 50 \end{array}\right]$$

The propagation and update are continuously calculated by the filter each time PPG sensor captures the heartbeat signal. In addition, Equation (13) can generate output of the information filter based on the updated inverse-mean (i.e., ${\hat{x}}_{k|k}$). In here, the filter output ${\hat{x}}_{k|k}$ is a matrix of 2 × 1 that can be used as a representative state of the variables in Equation (1), i.e., value ($a_{k}$) and rate of change ($b_{k}$):

(13) $${\hat{y}}_{k|k}=P_{k|k-1}^{-1}\;{\hat{x}}_{k|k}$$

After the filtering process, changes in heartbeat signal frequencies were analyzed using a fast Fourier transform (FFT) to get the RIIV distribution. The distribution was basically accumulated from a buffered FFT output at approximately 20 s intervals. Additionally, the distribution was updated for every data sampling period. Then, based on a peak-to-peak detection on the RIIV distribution, the respiration rate was calculated for each second.

## 3. Implementation of the Algorithm on an Embedded Device

**Figure 1:**
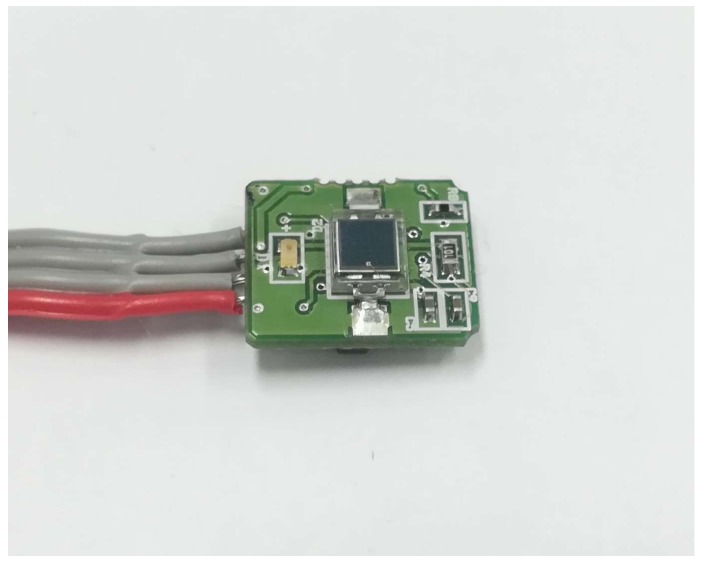
PPG Sensor Board, Laxtha RP520.

**Figure 2:**
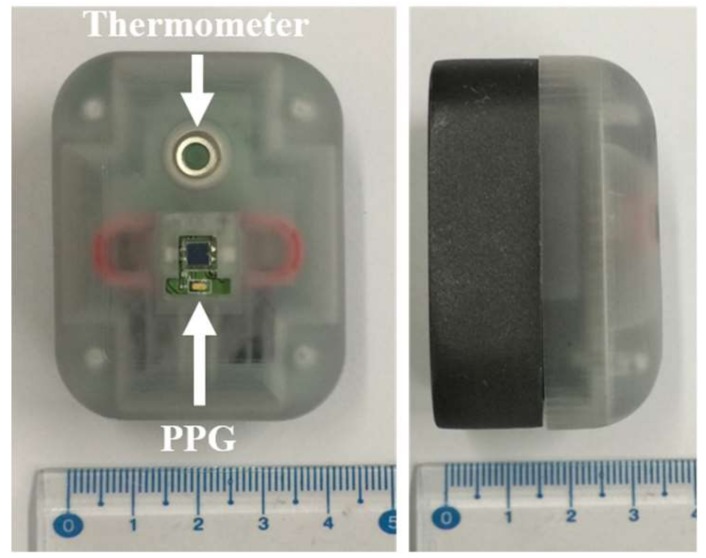
The embedded device for respiration rate measurement.

The embedded device consists of a PPG sensor, as shown in Figure 1, for heartbeat measurement, a thermometer sensor to facilitate body temperature measurement, and a CPU of an ARM processor, as shown in Figure 2. A Teensy 3.2 board was chosen as a base for the hardware development of the embedded device as the board has a small footprint with a 13-bit usable ADC and an ARM Cortex-M4 processor. The system can be used with a low-powered battery of 3.3~V with a CPU speed of 72 MHz. Using this system, the algorithm results can also be logged to other devices via serial communication.

The PPG signal in the embedded device was captured in a form of discrete number with noises via a 13-bit ADC system. Because the computational speed of the embedded device is relatively slow compared to a high-end computer system, the algorithm of the respiration rate measurement must use a simple process that can be implemented in such limited system. For that, the algorithm uses information filter to remove noises from the PPG signal as it has relatively light initialization process compared with Kalman filter. This filter is also chosen because sampling time of the PPG sensor readings is not always constant that brings noises and time inaccuracy to the PPG signal. In addition, only the value and rate of change of the PPG signal are filtered each time the sensor’s readings arrive. After that, the respiration rate is calculated based on observation of the filter’s output by using FFT analysis that conducted on a major peak only (i.e., frequencies with high value and occurrences).

**Figure 3:**
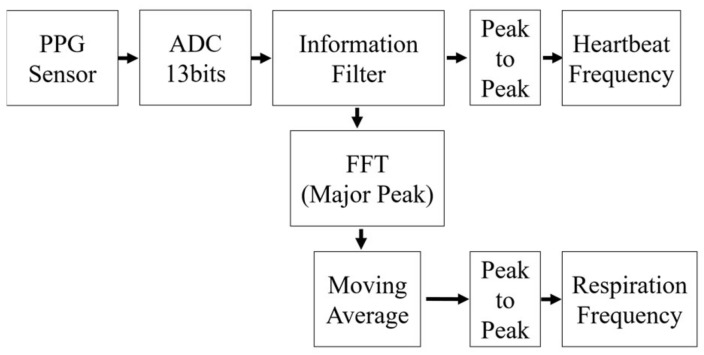
Simplified algorithm on the embedded device.

As shown in Figure 3, the PPG signal in the embedded device that was captured from a 13-bit ADC system and containing measurement noises, was approached by using an information filter. With this approach, the discrete numbers of the ADC reading can be linearized and the noises can also be reduced so that the signal can be observed more effectively by the FFT analysis.

**Figure 4:**
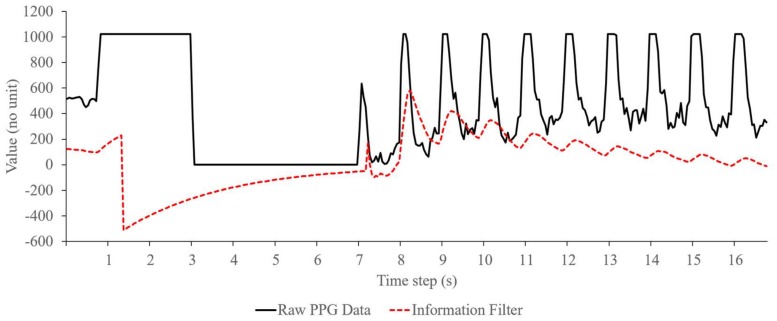
Typical signal filtering using information filter on the embedded device.

Figure 4 illustrates a typical output signal of the information filter. In this figure, raw data from the ADC system are displayed as a solid line and the filter output is distributed in a dotted line at each time step of approximately 50 ms. As shown in the figure, the PPG sensor took approximately seven seconds before it started measuring the heartbeat signal. During this time, the ADC output was raised to a maximum value and then significantly dropped non-linearly to zero (significant increase-and-decrease). On the other hand, the information filter output follow the ADC data linearly on every time step with delay of about 150 ms. Based on the figure, initial heartbeat detection will be calculated after a delay of about 1.5 s from the first detected heartbeat signal of the PPG sensor. This means that the algorithm has about a 1.5 s delay or initial detection time for heartbeat measurement.

**Figure 5:**
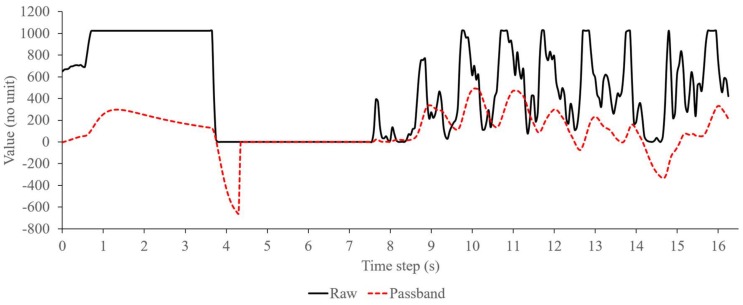
Typical signal filtering using a passband filter on the embedded device.

Compared with the passband filter that passes signal data within a range of the filter’s cutoff frequencies, the information filter has different criteria to validate the signal, i.e., based on the probability of mean and covariance of the filter’s estimation state and observation model. On the embedded device, the passband filter approaches the PPG signal that pass through the cutoff frequencies of a serialized low and high pass filter, as shown in Figure 5. Here, from 4 s to 7 s, the passband filter output (shown in dotted-line) immediately follows the raw signal data (in straight-line) from PPG sensor as the signal passes the filter’s cutoff frequencies. On the other hand, during the same time in Figure 4, the information filter slowly approaches the raw signal data because of the previous significant increase-and-decrease that lowers the probability of signal validity. Then after 7s, as the filter assures about the validity of the signal, it starts to follow the PPG signal similar with the passband filter. Therefore, when a fluctuation or distortion occurs on the PPG signal during the measurement time, the information filter will not immediately follow the signal before assuring its data validity. On the other hand, the passband filter will pass the signal as long as the signal is within its cutoff frequencies range, regardless uncertainties in the signal data. Based on this, the information filter on the embedded device can actually perform better than the passband filter for removing noises of the PPG signal, especially when the signal contains uncertainties or distortion that is usually caused by fluctuation of sampling time.

Once filtered, the frequencies of the PPG signal were evaluated by using an FFT analysis. In the embedded device, the FFT analysis was conducted on a major peak only and was directly measured in frequency domain. Since the FFT output can actually describe distortion in the input signal, changes of intensity in the heartbeat signal due to intrathoracic pressure can also be measured by monitoring the major peak’s variation. Then with a moving average filter and peak-to-peak detection, the respiration rate can be calculated based on these variations that show the changes in the filtered heartbeat signal [15,16,22,28].

Since the major peak’s variation can actually be observed in a discrete number, the output of the FFT analysis is calculated to that discrete number too. Thus, the FFT output could not describe the heartbeat rate accurately. Due to this, a peak-to-peak analysis was used to derive the heartbeat rate from filtered data, as shown in Figure 3.

**Figure 6:**
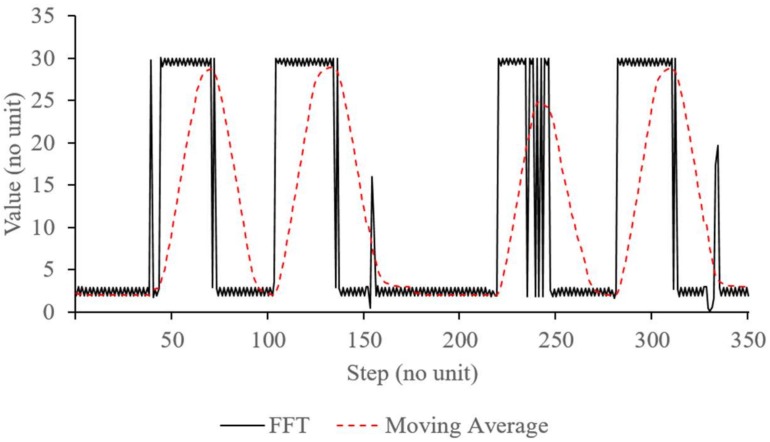
FFT and moving average signal.

Figure 6 shows the FFT analysis and moving average filter output. The solid line illustrates the major peak’s distribution at each time step. The moving average output that were calculated based on the major peak values are distributed as a dotted line. In here, the FFT analysis was configured with 64 data points and frequency resolution of 40 Hz. Additionally, the moving average filter was calculated based on 20 samples of FFT output with sampling time of 50 ms. As shown in the figure, when the major peak intensity is changed, the moving average value follows it linearly in a form of a signal peak. Therefore, in this figure, there are about four significant changes in about 300 time step which can be calculated as a respiration rate of about 0.27 Hz.

During implementation in the embedded device, the filter optimization was tested with two breathing conditions, i.e., a normal-relaxed breathing of a test subject sitting on a chair, and an induced-stress breathing of the test subject sitting on a chair after exercising. The exercise was performed by running on a treadmill with inclination level of 2% and a speed level of 5 for approximately five minutes. While the subject was still in recovery phase or post-exercise condition, measurement of heartbeat and respiration rate was immediately conducted for about 120 s.

## 4. Experiment and Results

**Figure 7:**
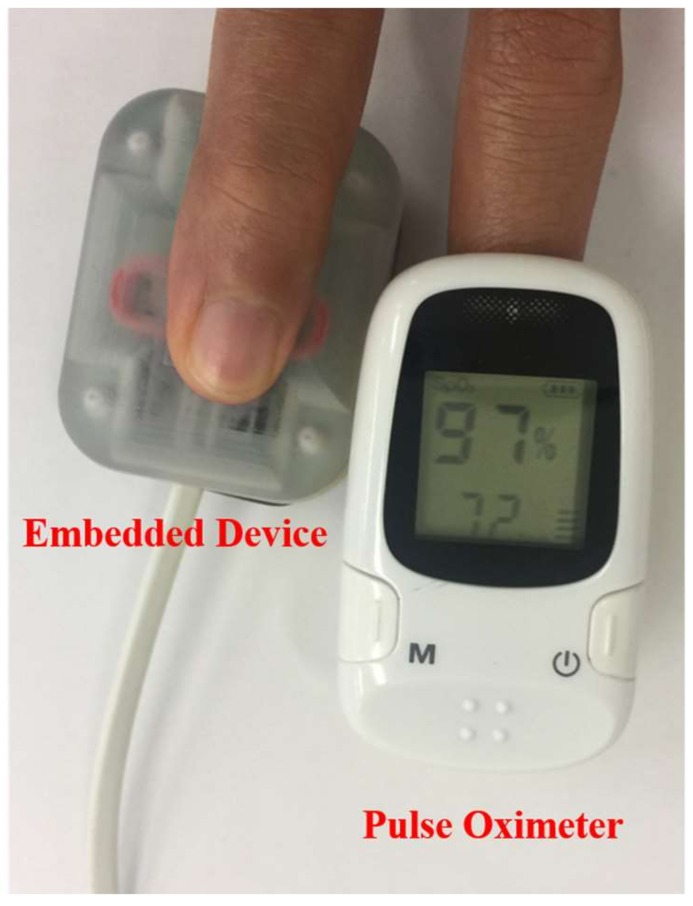
Heartbeat measurement setup with the subject’s hand.

For the heartbeat frequency calculation, a pulse oximeter was used to measure the heartbeat frequency of the test subject, as shown in Figure 7. The measurement outputs from the oximeter and embedded device were logged simultaneously during the test.

**Figure 8:**
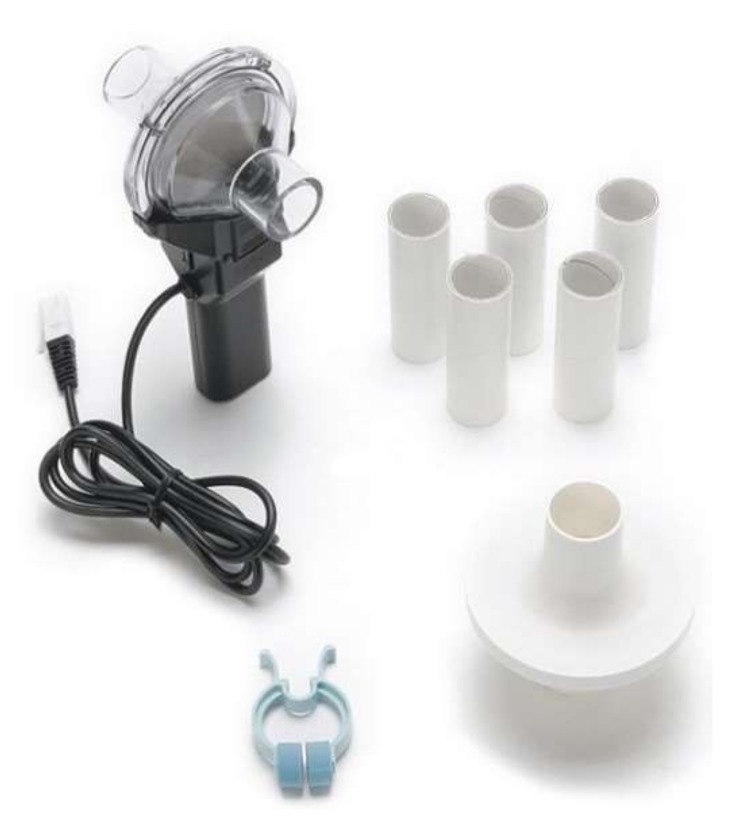
Vernier spirometer.

**Figure 9:**
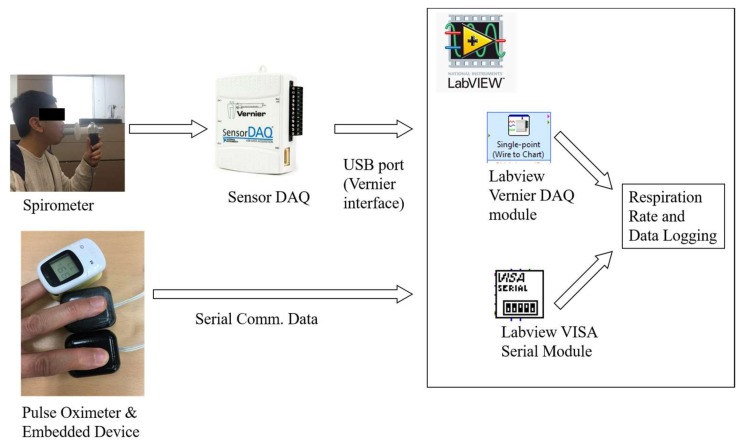
Heartbeat and respiration rate measurement setup.

Meanwhile, a spirometer (i.e., Vernier Spirometer with order code SPR-BTA) was utilized to validate the respiration rate algorithm, as shown in Figure 8. Here, the test subject held the spirometer in one hand and breathed through it, while the other hand was placed on the embedded device and pulse oximeter, as shown in Figure 9. The spirometer was connected to a Vernier SensorDAQ that could send the spirometer signal data to a computer or PC. During the test, the embedded device was also connected to the PC via serial communication for data logging only. All computation and algorithm processes were conducted using the embedded device. Data from the embedded device, spirometer, and pulse oximeter were then logged to a file using a LabVIEW interface.

This experiment had ten test subjects with an average age of 30 years and were healthy. For each subject, approximately 10 measurements were performed with one measurement time of about 5–10 min for normal relaxed breathing and about 120 s for post-exercise measurement. Between measurements, there was a 60 s time interval for resetting and stabilizing the measurement values of the oximeter and the embedded device. During this time interval, the oximeter and the embedded device were detached and reattached to the test subject’s finger. Then the differences between the algorithm results with the pulse oximeter and spirometer output were calculated to validate the heartbeat and the respiration rate measurement.

The measurement results of the embedded algorithm were evaluated for two breathing conditions (i.e., normal relaxed and post-exercise) using the pulse oximeter and spirometer. Based on these, performance of the embedded algorithm, especially for respiration measurement, was calculated for the two conditions.

The heartbeat measured by the algorithm in the embedded device was averaged at 79 bpm, as shown in Table 1. Based on the table, the heartbeat measurement value was similar to that of the pulse oximeter with a maximum difference of 5 bpm. In Table 2, the respiration rate was measured from 0.24 Hz to 0.30 Hz based on the embedded algorithm with a difference of 0.03 Hz in the average value.

Table 3 lists the measurement differences of the embedded device for the test subject during normal relaxed breathing and post-exercise. Here, the heartbeat rate difference is approximately 4.31% compared with that of a pulse oximeter output. On the other hand, the respiration rate difference is averaged at 8.12% compared with the spirometer results. This result shows that the simplification procedures and filtering process of the raw heartbeat signal were successfully implemented in the embedded device with reasonable average accuracies of about 90%.

**Figure 10:**
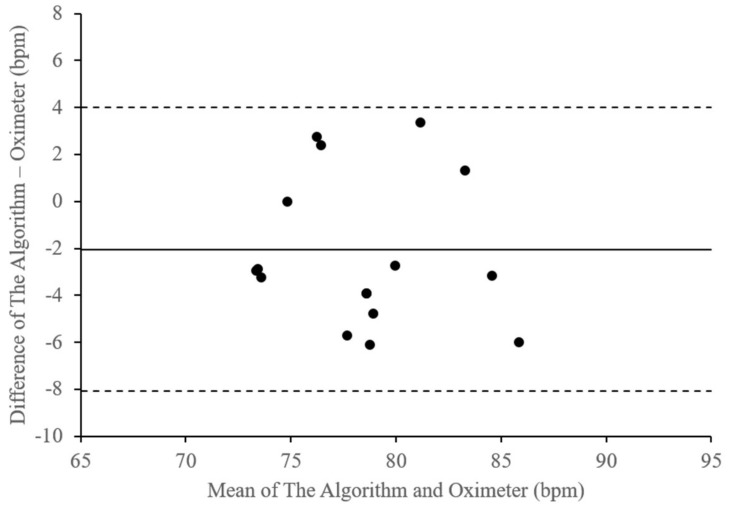
Bland-Altman Plot of Heartbeat Rate Differences (bpm).

Figure 10 illustrates these differences in the heartbeat rate measurement results of the embedded device compared with those of the pulse oximeter output. Table 4 lists the plot parameters of the heartbeat rate differences. The figure and table describe a bias of –2.03 bpm, with a lower limit of agreement of –8.06 bpm and an upper limits of agreement of 4 bpm. With that, the average of the difference is approximately 4.31% with a standard deviation of about 3.17.

**Figure 11:**
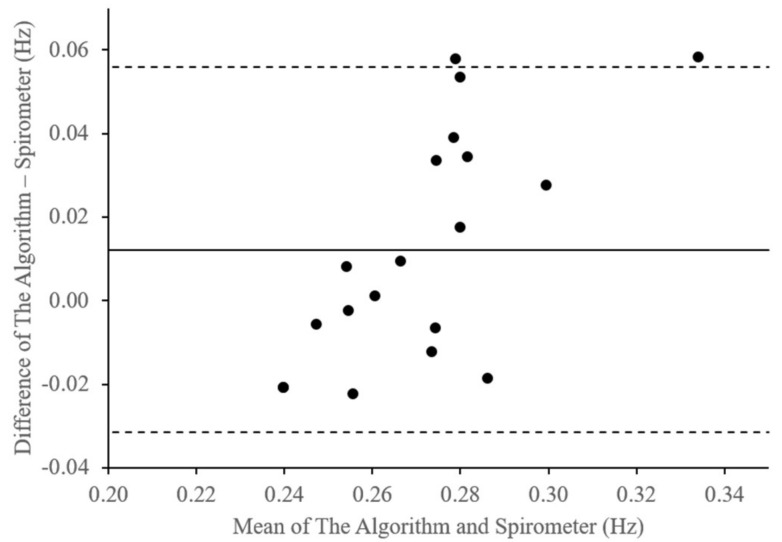
Bland-Altman plot of respiration rate differences (Hz).

Likewise, Figure 11 displays the differences in the respiration rate measurement results of the embedded algorithm compared with the spirometer for the two breathing conditions. As shown in Table 5, the bias is approximately 0.01 Hz with lower and upper limits of agreement of –0.03 Hz and 0.06 Hz, respectively. Additionally, the average difference is approximately 8% with standard deviation of about 0.02.

On the other hand, Table 6 shows differences between measurement results of the embedded device that utilizes a passband as the filter, compared with that of the pulse oximeter, for the heartbeat rate and spirometer, for the respiration rate. In here, the filter was implemented via serial of low and high pass filters due to the limited resources of computation power of the embedded device. FFT and other calculation process to derive respiration rate from the filtered data, were conducted with the same configuration as that in the embedded device with information filter. All measurement for the two embedded device, i.e., one with the passband and other with the information filter, were conducted simultaneously on two breathing conditions, i.e., normal and post-exercise breathing, as shown in Figure 9.

Based on Table 6 measurement differences of heartbeat rate are averaged at 4.15% with minimum and maximum differences of about 0.13% and 8.87%, respectively. In the respiration rate measurement, the average difference is about 9.05% with minimum and maximum differences of about 0.26% and 19.36%. Comparison of the two differences in Table 5 and Table 6 shows that average measurement difference of heartbeat rate of the embedded device with information filter are about 0.16% higher than that with the passband filter. On the other hand, the minimum and maximum measurement differences of heartbeat rate of the device with information filter are about 0.1% and 0.8%, respectively, lower than that with passband filter. In addition, the differences of respiration rate on the device with information filter are about 0.93% lower than that with passband filter. The maximum difference of the respiration rate measurement with information filter is also approximately 0.56% lower than that with the passband filter, which proves the effectiveness of the information filter.

In addition to a validation test, the initial detection and settling time of the algorithm in the embedded device with information filter was also calculated. Here, the distribution was calculated to observe the computational time of the algorithm implementation in the embedded device. The initial detection time was calculated between the first PPG signal detection and the first heartbeat and respiration rate calculation outputs.

**Figure 12:**
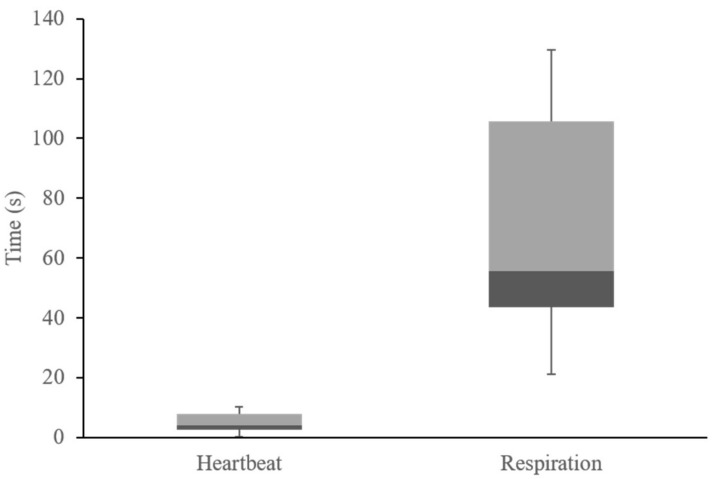
Heartbeat and respiration initial time distribution.

Figure 12 shows the average distribution of the heartbeat and respiration measurement initial times of the embedded algorithm. In this figure, the initial heartbeat detection time is averaged in about 4 s. The fastest time is approximately 0.33 s from the first heartbeat signal that was detected by the PPG sensor. On the contrary, the initial respiration detection time is about 53 s owing to the FFT buffering and sampling time. With that, respiration rate output values of the algorithm in the embedded device are converged in less than 90 s. As shown in Table 7, the fastest detection time is about 21 s.

Table 7 shows the initial detection time and measurement differences of the embedded device. Here, the heartbeat rate difference is approximately 4.31% compared with that of the pulse oximeter output. The average respiration rate difference was 8.12% compared with the spirometer for normal relaxed and post-exercise breathing conditions.

## 5. Discussion

Heartbeat and respiration rate measurement system was implemented on the embedded device by utilizing one PPG sensor module. Heartbeat rate was calculated based on peak to peak analysis of the PPG signal which was filtered using an information filter. The respiration rate was derived by monitoring variation in the filtered PPG signal using a simplified FFT analysis, directly in the time domain. With that the embedded device was equipped to a field robotic system which was deployed for search and rescue operation of a victim in a dangerous area.

On the embedded device, PPG signals is prone to error, such as number discretization of ADC system and sampling time fluctuation. In addition, other well-known sources of error are ambient light at the photodetector, poor blood perfusion of the peripheral tissues, and motion artifacts can lead to inaccurate interpretation of the PPG waveform [29,30,31]. In a dangerous area, these potential error sources can become a serious obstacle to the reliable use of PPG applications in a real-time application. However, the embedded device was concealed in a safe compartment of the field robot system and deployed after removing obstruction in the measurement area that can reduce the errors. In addition physical state determination (i.e., heartbeat rate and respiration rate) was conducted for a victim in a critical condition which was highly assumed to be unconscious or unable to move. 

Unlike [31] that used pattern recognition to extract PPG waveform, an FFT analysis was implemented to monitor variation of the PPG signal. Due to limited resources of the embedded device, FFT analysis was simplified and conducted in time domain, similar with [30]. Adding moving average to the FFT can extract useful filtered signal data that correspond to the occurrences of RIIV as shown in Figure 6. With this, respiration rate can be derived relatively accurately based on the peak-to-peak analysis of the output of the moving average calculation.

## 6. Conclusions

An algorithm to measure the respiration rate was successfully implemented in an embedded device. The device utilized a PPG sensor to capture heartbeat signals, which were then filtered using an information filter. Then, an FFT analysis was conducted to calculate the respiration rate based on RIIV observations of the filtered data. Here, the algorithm can measure the heartbeat and respiration rate with bias of −2.03 bpm and 0.01 Hz, respectively. In addition, based on test results of 10 subjects with two breathing conditions (i.e., normal and post-exercise breathing), the algorithm computation had standard deviation of 3.17 for the heartbeat measurement and 0.02 for the respiration rate.

Based on these results, it can be concluded that the algorithm in the embedded device can measure the heartbeat rate with a maximum of approximately 10% difference or approximately 90% accuracy. On the other hand, the delay in the computational process is approximately four seconds from the first detected heartbeat signal by the PPG sensor, with an additional seven second for the sensor initialization. Then, for normal and post-exercise conditions, the embedded device can measure the respiration rate with an average difference of approximately 8% or approximately 90% accuracy. Additionally, the maximum difference is approximately 19% (or an accuracy of approximately 80%) compared with the spirometer. Moreover, comparison of the measurement differences between two filters, i.e., passband and information filters show that the maximum measurement differences of the two vital signs (i.e., heartbeat and respiration rate) of the embedded device with the information filter are smaller than that with the passband filter, which proves the effectiveness of the information filter in the embedded device.

## 7. Future Works

In this study, the heartbeat and respiration rate measurements were conducted on a conscious person in a stable environment. The algorithm for the embedded device was successfully implemented with a reasonable measurement value compared with other medical devices. For general use and future development, we will attach the embedded device to a manipulator and test it in a different environment. In addition, the measurement and test of the embedded device will be conducted under varying test conditions, such as age or body posture, on an unconscious or sleeping subject. Moreover, spontaneous and controlled breathing are expected to be observed as they tend to affect the PPG sensor readings, especially those for RIIV measurements [22].

## Figures and Tables

**Table 1:** Comparison of heartbeat (bpm) measurement between pulse oximeter and embedded algorithm.

Method/Tool	Min	Average	Max
Pulse Oximeter	58	77	84
Embedded Algorithm	57	79	89

**Table 2:** Comparison of respiration rate (Hz) measurement between spirometer and embedded algorithm.

Method/Tool	Min	Average	Max
Pulse Oximeter	0.22	0.29	0.36
Embedded Algorithm	0.24	0.26	0.30

**Table 3:** Differences of the algorithm in embedded device.

Parameter	Min	Average	Max
Heartbeat Rate Diff. (%)	0.03	4.31	8.07
Respiration Rate Diff. (%)	0.43	8.12	18.8

**Table 4:** Parameter values of heartbeat rate differences (bpm).

Parameter	Value
Bias	−2.03
Standard Deviation	3.17
Upper Bound	4.00
Lower Bound	−8.06

**Table 5:** Parameter values of respiration rate differences (Hz).

Parameter	Value
Bias	0.01
Standard Deviation	0.02
Upper Bound	0.06
Lower Bound	–0.03

**Table 6:** Differences of the algorithm in embedded device using the passband filter.

Parameter	Min	Average	Max
Heartbeat Rate Diff. (%)	0.13	4.15	8.87
Respiration Rate Diff. (%)	0.26	9.05	19.36

**Table 7:** Differences of the algorithm in embedded device.

Parameter	Min	Average	Max
Initial Heartbeat Detection Time (s)	0.33	3.81	7.43
Initial Respiration Detection Time (s)	21.14	53.32	86.14
Heartbeat Rate Diff. (%)	0.03	4.31	8.07
Respiration Rate Diff. (%)	0.43	8.12	18.8
